# Honeycomb-like Ag Nanocavity Array for SERS Observations Using Plasmon-Mediated Chemical Reactions

**DOI:** 10.3390/mi14101811

**Published:** 2023-09-22

**Authors:** Yongjun Zhang, Zhen Xu, Jiahong Wen, Xiaoyu Zhao, Renxian Gao, Yaxin Wang

**Affiliations:** 1 School of Material and Environmental Engineering, Hangzhou Dianzi University, Hangzhou 310018, China; yjzhang@hdu.edu.cn (Y.Z.);; 2 The College of Electronics and Information, Hangzhou Dianzi University, Hangzhou 310018, China; 3 Zhejiang Laboratory, Hangzhou 311100, China; 4 Department of Physics, Xiamen University, Xiamen 361005, China; renxiangao2020@163.com

**Keywords:** LSPR, PMCRs, Ag growth, SERS

## Abstract

Organized two-dimensional polystyrene bead arrays perform ion etching, and protruding nanostructures are created on polystyrene beads due to the shadow effects from the ring beads, leading to nucleus selection and growth in Au nanostructure deposition. Ag nanostructures are prepared via plasmon-mediated chemical reactions (PMCRs), leading to the Ag nanocavity geometry of the honeycomb pattern when the etching time and Ag growth time are tuned. Due to the strong electromagnetic coupling, the Ag honeycomb-shaped nanocavity array works as the SERS substrate with high sensitivity and good repeatability, which is used to detect thiram pesticide residues with a concentration down to 10^−9^ M.

## 1. Introduction

To provide more food and prevent crop loss, various pesticides are frequently employed in modern agriculture to prevent plant diseases, control insect pests, and encourage plant growth. When pesticides are used improperly, they can persist in the environment and built up in food, eventually becoming harmful to human health [1]. It has been established that thiram, a commonly used antibacterial pesticide, builds up in the human body and harms the skin and mucous membranes. Although the guidelines for its usage have been approved, it is difficult for regular consumers to quickly and accurately detect thiram residues [2]. Therefore, there is an urgent need for quick and easy techniques for the detection of thiram residues in our daily life.

In recent years, observations related to surface plasmon polaritons in nanostructures have attracted the attention of many researchers because of their important theoretical significance and widespread application, including the detection of chemical molecules with a low concentration [3]. According to classical physics, the coherent motions of electrons, which are fueled by the electromagnetic field of incident light, carry a significant amount of energy before relaxation. This energy can be transferred into the environment through the relaxation process, which increases the local energy surrounding the nanostructures [4]. In this process, the plasmonic nanostructures can capture incident light throughout the entire visible spectrum range and convert it into photoelectricity when different shapes and materials are chosen. Local surface plasmon resonance (LSPR) excited in nanostructures can enable the nanostructures to collect the energy of incident light and strongly enhance the electromagnetic field at the tips, gaps, and cavities, which are referred to as hotspots. Hotspots are regions of high local energy on the nanoscale that occur when nanostructures are close to one another, and the local electromagnetic fields tend to couple together [5]. At the same time, the energy of the plasmonic field can cause the metal to generate hot carriers, which are dissipated by interacting with phonons in the metal nanostructures, leading to an increase in lattice temperature [6]. These nanoplasmonic materials, as the effective sources of electrons, light, and heat, can greatly promote the development of the field. Therefore, when incident light excites noble metal nanostructures to generate plasma resonance, it will enhance the electromagnetic field, generate hot carriers and thermal effects, and promote chemical reactions between the surface substances of plasmonic nanostructures, which can be controlled at the nanoscale [7]. In recent years, with the development of the preparation and characterization technology of nanostructures, researchers have designed and prepared various types of micro/nanostructures, which find widespread applications in the fields of sensing [8], medical diagnosis [9], electromagnetic stealth [10], photocatalytic degradation [11], negative refraction [12] and surface-enhanced Raman scattering [13].

Numerous characteristics of nanostructures, such as the separation of materials, the shape, the chemical composition, and the nanostructure itself, affect the energy and the dispersion of hotspots [14]. These parameters are also the adjustable options used to obtain desired hotspots. Generally speaking, the methods used to prepare nanostructures are lithography and electron beam lithography (EBL) in most cases, but the complex preparation processes and expensive equipment involved increase the cost of these experiments [15]. In addition to these two methods, nanotechnology based on nanosphere lithography [16] is more popular because of its simple operation and low cost. However, some significant shortcomings such as low repeatability of the substrate structure and instability of the substrate structure are often mentioned with regard to nanosphere lithography, which drive the extensive efforts of researchers regarding the development of new materials for detection, sensing, and other technologies.

In addition to sensing, detection and other applications, noble metal plasmonic nanostructures can also be used as a new type of photocatalyst for chemical reactions, namely plasmon-mediated chemical reactions (PMCRs) for material synthesis [17]. Under illumination, plasmonic nanoparticles are also excited, leading to collective oscillations of conductive electrons. If the excitation frequency matches the free electron oscillation frequency, local surface plasmon resonance will occur, resulting in significant electromagnetic enhancement [18]. A certain portion of the energy is stored in the plasmon excitons, which can be re-emitted as the photons when the surface plasmons relax, and these photons can decay to produce electron–hole pairs [19]. If these hot carriers of high energy can be effectively extracted from plasmons, they can effectively reduce the activation barrier of chemical reactions and trigger chemical reactions which are not supported by traditional thermodynamics or kinetics [20]. The spontaneous formation of ordered structures during the growth of nanocrystals can be used to prepare nanostructures with different shapes or periodic arrays [21]. This method can solve the aforementioned problems and be widely applied in fields such as biosensing, detection, and anti-counterfeiting [22]. Plasmonic photocatalysts can drive photochemical reactions in the spectrum in the range from the ultraviolet to the visible and to the infrared regions, which widens the application fields of traditional photocatalysis [23]. The plasmonic effects of the collective electrons are controlled by the coherent oscillation of free electron gas in metal nanoparticles at the frequency of the incident electric field. If the frequency of the incident light matches the local surface plasmon resonance frequency, it can further enhance the excitation of these modes, promoting chemical reactions. This process is accompanied by meaningful physical effects such as field effects, thermal effects and high-energy carriers, which can be used in chemical reactions, promote chemical reactions and limit reaction products to specific areas, so as to achieve localized reactions of the chemical process on a nanoscale, referred to as plasmon-mediated nanochemistry [24]. Plasmonic nanomaterials, as the effective sources of electrons, light, and heat, can greatly promote the development of this field. The key parameter for PMCRs is how to realize the control of chemical reactions on metal surfaces for different applications. Tijunelyte et al. investigated the PMCRs near gold nanostructures in different conditions, which demonstrated that this reaction could be controlled using the plasma tuning properties of nanostructures [25]. Emiliano Cortés et al. pointed out that plasmon-assisted chemical growth was the result of the complex interaction between the electromagnetic field, heat, and charge transfer on a nanoscale, which meant that the final product in nanochemistry reactions could be controlled by adjusting the electromagnetic coupling and hotspot distribution [26]. Zhan et al. introduced the similarity and distinctive features of plasmon-enhanced molecular spectroscopy (PEMS) and plasmon-mediated chemical reactions (PMCRs) and compared PMCRs with traditional photochemical and thermochemistry reactions, which explained how to improve PMCRs by reasonably designing and manufacturing plasmonic nanostructures and selecting suitable surface and interface mediators [27]. Wang et al. summarized the latest plasmonic nanostructures manufactured through colloidal photolithography and classified them into multi-layer, layered, hollow, asymmetric, and other nanostructures in morphologies [28].

PMCRs is a new field of research and development which redistributes and converts photon energy into local photon, electron, and thermal energy by using plasma nanomaterials as the media [29]. In addition, based on the unique properties of plasma nanomaterials, PMCRs show potential advantages differing from traditional thermochemistry, photochemistry and photocatalysis. PMCRs find widespread applications in many fields, such as biological imaging [27], biosensors [30], drug delivery [31], and photothermal cancer therapy [32]. Zhan Chao et al. used surface-enhanced Raman spectroscopy to reveal plasma-mediated chemical reactions in nanostructures and the correlation between PMCRs and SERS [33]. Guan et al. prepared Ag nanoparticles solely by observing the maximum plasmonic field areas of Au nanohole arrays under light illumination, and Ag nanoparticle-assembly arrays with controllable morphology ranging from nanorings to nanodisks were realized, providing a new method for creating anti-counterfeiting tags for electronic devices [34]. Zhu et al. prepared Ag nanoparticle arrays with different sizes and positions using PMCRs in a Au nanobowl array, and the growth of six axisymmetric and triaxial periodic Ag nanostructures was realized by using circularly polarized light and linearly polarized light, which realized the tunable fabrication of the different patterned nanostructures [35]. Since Alak and Vo Dinh used SERS spectroscopy to detect pesticides in 1987, people have begun to study the application of SERS spectroscopy in pesticide detection extensively [36]. Guo et al. prepared samples of Au@Ag with a core–shell structure with different particle sizes and morphologies, which realized the detection of thiram pesticide based on the analysis of SERS spectra [37]. Sun et al. reported a green and environmentally friendly PMMA/AgNP/graphene composite structure, which was also used as a SERS-active substrate suitable for the in situ monitoring of trace thiram in apple juice with a detection limit of 1 × 10^−6^ M [38]. Nguyen Ha Anh et al. deposited gold nanoparticles on an aluminum substrate electrochemically, which was utilized to monitor thiram in complicated conditions such as in food samples as a SERS sensor [39].

We prepared a honeycomb-like Ag nanocavity array using polystyrene colloidal spheres as a template, combined with plasma etching, physical deposition and plasma-mediated chemical reactions. For compact PS nanosphere arrays, the ion etching process show a slow etching rate between the PS nanospheres, which is referred to as the shadow effect. By controlling the time of plasma etching, microspheres with synaptic structures are obtained via the shadow effect of adjacent polystyrene pellets. After physical deposition and chemically assisted growth, the silver nano-honeycomb structure is finally obtained. Finite-difference time-domain (FDTD) simulation is used to obtain the distribution law of hotspots, which indicates the abundant hotspots in this honeycomb-like Ag nanocavity array. This honeycomb-like Ag nanocavity array is chosen as an active substrate for the detection of a low concentration of the pesticide thiram, at 10^−9^ M.

## 2. Materials and Methods

The polystyrene (PS) dispersion (10 wt%, diameter 500 nm) was purchased from Duke Co., Ltd. (Palo Alto, CA, USA). Aladdin (Beijing, China.) provided 4-mercaptobenzoic acid (4-MBA, 99%), thiram (99%), silver nitrate (99.7%), and sodium citrate solid particles. Beijing Tianqi (Beijing, China) Advanced Materials Co., Ltd. (HZTQ) supplied the Au (99.999%) target for magnetron control sputtering. Hefei Kejing Materials Technology Co., Ltd. (Hefei, China) supplied the silicon wafers. The Millipore water filtration system provided the deionized water (18.2 MΩ·cm^−1^).

A PS monolayer film was self-assembled on a silicon wafer at the liquid–gas interface, as previously described [40,41,42]. In brief, the hydrophilic silicon substrate was obtained via boiling the silicon wafer for 20 min in a solution of ammonia, hydrogen peroxide, and deionized water (volume ratio 1:2:6). The polystyrene bead solution and absolute ethanol (volume ratio 10:7) solution were sonicated for 5 min to fully mix them before being added to the surface of the deionized water to form a dense monolayer. The cleaned silicon wafer was used to pick up the PS monolayer and was exposed to oxygen reactive ion etching (RIE, 20 Pa, 50 sccm, 50 W). The magnetron control sputtering system (modeled ATC 1800-F, AJA, Scituate, MA, USA) was used to deposit Au films. The base pressure for magnetron control sputtering was 2 × 10^−6^ Pa, while the pressure during sputtering was 0.6 Pa. The power was 50 W, and the oxygen and nitrogen flow rates were both 20 sccm. The target plate and the substrate were separated at a distance of 10 cm. The sputtering time was set to 2 min and the power was set to 10 W. Ag nanoparticle growth was triggered by PMCRs in a mixed solution of sodium citrate and silver nitrate under natural light. Au nanostructures were immersed in a mixed solution of silver nitrate (0.5 mM/100 mL) and sodium citrate (25 mM/6 mL) under natural light irradiation. For FDTD simulation using Lumerical FDTD Solutions, the nano-honeycomb was built in the X–Y plane and perpendicular to the X–Z plane. The dielectric constant and permeability values of Ag were obtained in the FDTD database. The refractive index of 1.585 was set for PS spheres. The PS sphere radius was 500 nm, with the distances obtained in SEM images after etching. For different etching times, the PS sphere radius and the distance between nanorods changed. A grid size of 1 nm was chosen, and a conformal variant was set to 2 for mesh refinement. The simulation time was 1000 s overall, and the minimum value for auto shutoff was set as 1 × 10^−5^ with a simulation temperature of 300 K.

A field emission scanning electron microscope (15 kV, JEOL 7800F, Tokyo, Japan) was used for morphology characterizations. The Raman and SERS spectra were collected on a Renishaw Raman system model 2000 confocal microscopy spectrometer equipped with a charge-coupled device detector and a holographic notch filter. The wavelength of the light source was 632.8 nm with a laser beam spot diameter of 1 μm, and a 50× long-range objective was chosen. The laser power was 17 mW and typical exposure time for each measurement was 10 s with a one-time accumulation. For each spot, three measurements were performed. The laser power attenuation was set to 1%.

## 3. Results

**Figure 1:**
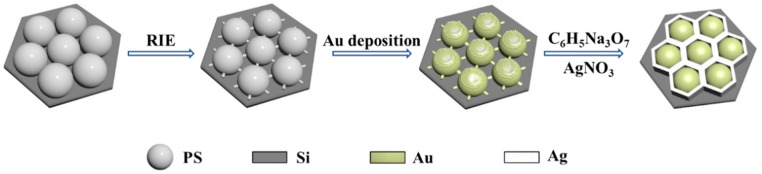
Schematic of the preparation of the Ag nano-honeycomb structure.

Figure 1 shows the fabrication process for a Ag nano-honeycomb structure using the PMCR method. The monolayer polystyrene (PS) colloidal sphere templates are prepared on silicon wafers via gas–liquid interface self-assembly. Then, the ion etching is carried out to separate the beads with certain distances. Due to the shadow effect from the neighboring beads, small protruding nanostructures are observed for each PS bead upon Au deposition, which work as the nucleating position for the subsequent Ag growth driven by PMCRs. Ag growth is carried out for different time periods and the Ag nanocavity array with a honeycomb-shaped pattern is created, which is suitable as a SERS substrate, due to the strong electromagnetic coupling.

**Figure 2:**
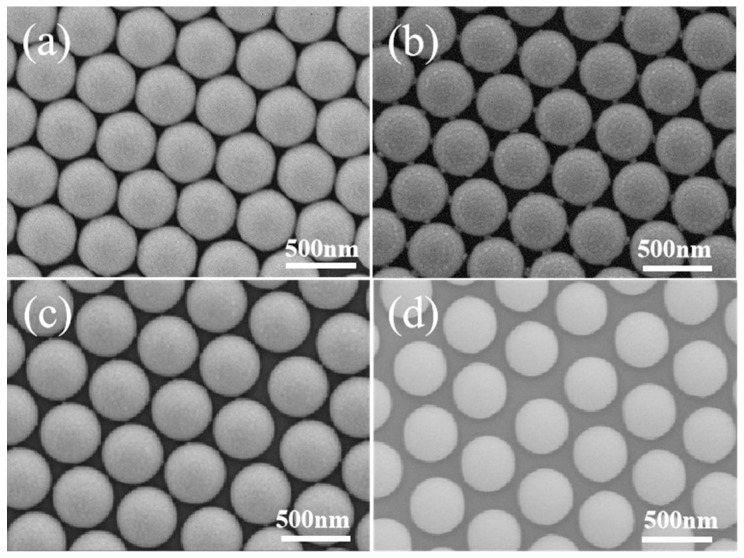
SEM images for PS beads etched for (**a**) 0 s, (**b**) 30 s, (**c**) 60 s, (**d**) 90 s.

The surface morphology of PS beads changes with the etching duration when the tightly packed PS array is subjected to ion etching, as shown in Figure 2. The connections between the neighboring polystyrene spheres are plainly visible when the etching duration is 30 s due to the shadow effect, and there is a 20 nm gap between the neighboring PS beads. In addition, there are six synaptic structures around each PS bead, which is due to the shadow effect from six neighbors. The space between two neighboring PS beads grows to 40 nm for beads etched for 60 s, and the size of the synaptic structure is decreased, indicating the reduced shadow effect due to the large separation between the neighboring PS beads. When the etching time is 90 s, the diameter of the polystyrene colloidal sphere decreases continuously. The synaptic structure disappears completely, and the distances between the neighboring beads are around 50 nm, as shown in Figure 2d.

**Figure 3:**
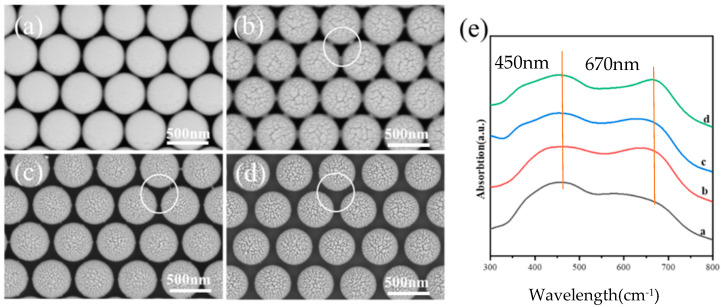
SEM images of polystyrene colloidal spheres processed using a plasma cleaning machine with etching times of (**a**) 0 s, (**b**) 30 s, (**c**) 60 s and (**d**) 90 s; (**e**) absorption spectra of Au films deposited on four different sizes of PS microspheres. The white circles show the changes of the protruding parts with the etching time.

When a Au film with a 10 nm thickness is deposited onto the PS beads after etching for 0–90 s, the surface morphologies change with the sizes of the PS beads, as shown in Figure 3a–d, which confirms the curvature’s effects on Au growth. For the PS array before etching, a smooth surface is observed, which indicates the continuous film deposited on PS beads. For the film deposited on the PS array after 30 s of etching, the curved Au film shows obvious roughness. The detailed analysis shows that this roughness is composed of many aggregated nanoparticles. In addition, the aggregations show a large size on top of the PS and the aggregation sizes decrease along the curved surface of the PS beads. Au film is also observed to grow on the synaptic structures as guided by the circle. For the film deposited on the PS array after 60 s of etching, the curved Au film shows roughness composed of aggregations with a small size in comparison to those grown on the PS array after 30 s of etching. For the film deposited on the PS array after 90 s of etching, the aggregations become small, and the nanoparticle density also became low. All the observations above indicate that small PS beads with a large curvature lead to small Au nanoparticles. At the same time, these Au nanoparticles show a more scattered distribution on small PS beads because the large curvature results in significant self-shadow effects due to the curved surface. Figure 3e shows the absorption spectra for Au films deposited onto the PS array after different etching times. The peak around 450 nm is due to the Bragg diffraction, because of the light scattering caused by the periodic structures. The sizes of the PS beads and the film have a broad distribution, resulting in the broad peak. The peak position around 670 nm originates from the local surface plasmon resonance of the Au nanostructures, and the shifts originate from the microstructure changes.

**Figure 4:**
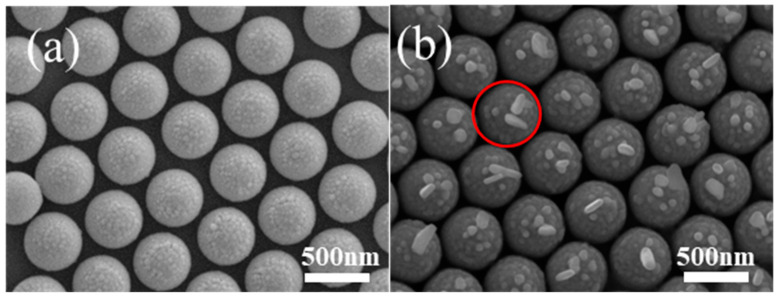
When a Au film with 10 nm thickness is deposited onto PS 500 nm after etching for 90 s, Au nanoparticles are observed only on the tops of PS (**a**), and the following Ag nanoparticle growth happens only on the tops of PS when PMCRs are performed for 30 min (**b**). The red circle shows the fast growth of Ag nanostructures by PMCRs near the large Au nanoparticles.

Au nanostructures can support surface plasmons, which can induce Ag nanoparticle growth. To perform the plasmon-induced chemical reaction, red light with an appropriate wavelength range is selected as the excitation light source. Before Ag growth, Au nanoparticles are observed around the top of the PS. When PMCRs are performed for 30 min, notable Ag growth is observed around the tops of the PS beads only, as shown by the red circles, in agreement with Au nanoparticle distribution, which confirms Ag growth according to the PMCRs mechanism, as shown in Figure 4. Some large Ag nanostructures are observed around large Au nanoparticles on the tops of PS beads, indicating strong electromagnetic coupling. In addition, some Ag nanostructures with a large aspect ratio are also observed, indicating the optimal growth induced by large Au nanoparticles. Au nanoparticles have a very small size around the nanocap brim, which cannot support the significant LSPR. Therefore, no Ag nanostructures with a large size are obtained away from top of the PS. For this patterned substrate, no coupling happens between the neighboring PS beads covered by Au nanoparticles because of the large spaces between the neighboring units. Therefore, Au nanoparticles on the PS beads show coupling of arbitrary symmetry, which leads to the random growth of Ag nanostructures as observed above.

The nucleation and growth of Ag nanoparticles not only depend on the photoreduction of Ag ions by citrate ions in the mixed solution, but also require enough free energy to realize the phase transition and movement of Ag ions into the Ag lattice for growth. The free energy ΔG of the Ag nanoparticles, which are formed when citrate ions convert Ag^+^ ions into AgNPs, is crucial for the nucleation and development of silver nanoparticles. Generally, ΔG is composed of two parts—the free energy for phase transformation ΔG_V_ related to the volume of nanoparticles and the free energy for a solid surface ΔG_S_ connected to the surface area of nanoparticles. In the physical view, only when ΔG is larger than the critical energy ΔG_critical_ for phase transition does the nucleus develop into a large and stable particle and maintain the growth. Therefore, the large electric field in the hotspot provides additional energy to ΔG, which means that earlier nucleation and faster growth occur around the hotspots. By designing LSPR to overcome the critical energy ΔG_critical_ at certain locations, the chemical reaction can be tuned to take place first at the nanometer scale, so that the self-assembly of nanoparticles can be completed during the synthesis process, which is called “in situ chemical patterning”. Wang et al. induced the growth of gold and silver nanoparticles in an aluminum inverted hollow nanocone cavity. By partially suppressing the light of IHNA, various levels of patterns were obtained at the macro, micro, and submicron scales, stimulating a simple patterning technique by changing the light source [28]. Guan et al. used this method to prepare silver nanoparticles based on the maximum plasma field area of the gold nanopore array under light conditions. The SERS performance also changed, with their assembly array achieving a controllable morphology of silver nanoparticles, from nanorings to nanodisks, which also provided a new method for creating anti-counterfeiting labels for electronic devices [21].

**Figure 5:**
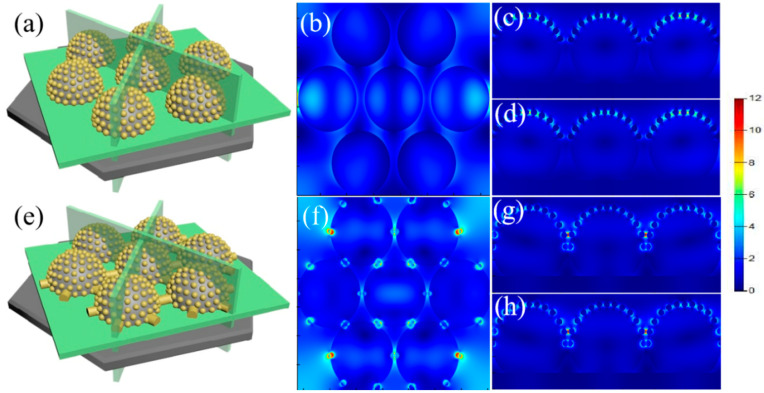
(**a**–**d**) FDTD simulations show that the enhanced electromagnetic field occurs between Au nanoparticles on the tops of the PS when a Au film with a 10 nm thickness is deposited onto the PS beads etched for 90 s; (**e**–**h**) FDTD simulations show that the enhanced electromagnetic field occurs between Au nanoparticles on the tops of the PS when a Au film with a 10 nm thickness is deposited onto the PS beads etched for 30 s.

To determine the exact mechanism for the production of Ag nanoparticles, the hotspot distribution is simulated using the FDTD algorithm, as shown in Figure 5. The wavelength of the light source is adjusted at 633 nm. The optimal geometry of the array of PS microspheres coated with periodic Au nanoparticles in the FDTD simulation is depicted in Figure 5. In other words, the simulation accurately predicts how hotspots will be distributed among the Au nanoparticles on the array of Au nanospheres. When there are no synaptic structures between the adjacent Au nanospheres, the hotspots are only found between the Au nanoparticles around the PS bead tops. In addition to the enhanced localized fields around Au nanoparticles, the large electromagnetic couplings are also found between Au nanoparticles. Due to the decreased sizes of Au nanoparticles along the surface of PS beads, the local field also decreases gradually, which means the decrease in the coupled electromagnetic field along the PS bead surfaces. The large coupling field supports the priority nucleation and growth at these locations, supporting the formation of large Ag nanoparticles on PS bead tops. When the PS beads with synaptic structures are used for Au deposition, Au nanostructures created on the synaptic structures also provide additional hotspots in addition to those on the tops of the PS. For PS etched for 30 s, the Au nanostructures created on the synaptic structures show much strong electromagnetic coupling than that of the Au nanoparticles on the tops of the PS, which indicates that the priority nucleation and fast growth occur near the synaptic structures in comparison to those on the tops of the PS.

**Figure 6:**
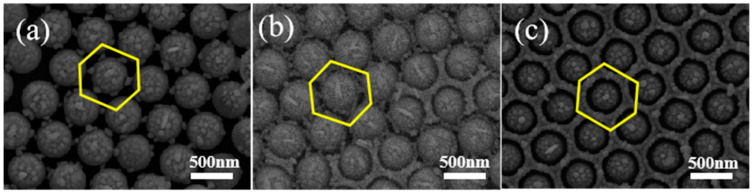
PMCRs are performed for different times on the PS colloidal bead array with an etching time of 30 s, and the honeycomb-like pattern comes into formation gradually. (**a**) 30 min, (**b**) 60 min and (**c**) 90 min. The yellow hexagons show the gradual formation of the honeycomb patterns.

For PS colloidal beads with an etching time of 30 s, the PMCRs are conducted in a solution that contains equal parts silver nitrate and sodium citrate, with the reaction time being 30 min to 90 min. When the PMCRs are carried out for 30 min, Ag nanoparticles are observed both on the tops of the PS and near the synaptic structures with sizes of about 20 nm, as shown in Figure 6a, which confirms the growth mechanism of Ag nanoparticles induced by PMCRs. When the chemical reaction is performed for 60 min, the number of Ag nanoparticles increases, and the Ag nanoparticles form a loose ring-like shape around the PS beads, as shown by the yellow ring in Figure 6b. At some locations, the loose Ag ring becomes a solid side wall between the neighboring PS beads. When the chemical reaction is prolonged to 90 min, a complete honeycomb-like pattern composed of Ag nanoparticles is formed, as shown in Figure 6c. The sizes of the Ag nanoparticles are about 50 nm. When LSPR is excited in this complete honeycomb-like pattern, the electromagnetic enhancement comes not only from the Au nanoparticles and the coupling between Au nanoparticles, but also from the coupling effects between Au nanocaps and the side wall. At the same time, the material transfer process is important for Ag growth, favoring Ag nanoparticle growth vertically and transversely to form the honeycomb-like pattern, due to the limited space between the Au nanocaps.

**Figure 7:**
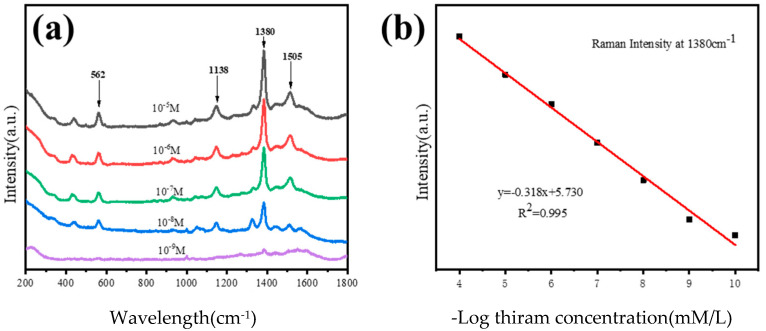
(**a**) SERS spectra for thiram at various concentrations found using the nano-honeycomb structure’s SERS substrate; (**b**) the SERS peak at 1380 cm^−1^ shows the linear dependence on the negative logarithm of different concentrations of thiram.

Our nano-honeycomb structures show arbitrary axis symmetry when chosen as the SERS-active substrate for the detection of thiram, as shown in Figure 7. The maximum residual level for thiram in food should not exceed 15 ppm, according to the most recent amendment by the US Environmental Protection Agency (about 10^−6^ M). Therefore, thiram with concentrations of 10^−5^ M, 10^−6^ M, 10^−7^ M, 10^−8^ M and 10^−9^ M is characterized by this SERS-active substrate. Under the excitation of a 633 nm wavelength, the characteristic peaks of the thiram SERS spectra are observed in Figure 7a. The Raman bands are attributed as follows: the stretching vibration mode of CN and the symmetry deformation of CH3 together produce the strongest peak in the SERS spectra at 1380 cm^−1^, the peak at 1438 cm^−1^ belongs to the antisymmetric stretching mode of n(CH3), the peaks at 1138 cm^−1^ and 1505 cm^−1^ correspond to the stretching vibration of CN and the swing mode of CH3, and the stretching vibration mode of S-S is represented by the peak at 562 cm^−1^ [23]. The quantitative analysis of thiram concentrations is performed using the peak at 1380 cm^−1^ for the samples from 10^−5^ M to 10^−9^ M, which can be fitted according to the linear function y = −0.318x + 5.730. The correlation coefficient is calculated as 0.995, which indicates that the function and the fitting process work for our analysis (Figure 7b).

## 4. Conclusions

The honeycomb-like nanostructure array is prepared based on an ordered PS template by means of plasmon-mediated chemical reactions (PMCRs). The PS template is composed of 500 nm PS beads, which are processed via ion etching to obtain the tunable distance and surface morphologies. Due to shadow effects, synaptic nanostructures may be seen when the etching period is 30 s, which encourages the formation of Au nanostructures during the subsequent deposition of the Au film with a 10 nm thickness. FDTD simulations show that strong electromagnetic fields are observed between the nanoparticles at the top of the PS and the synaptic nanostructures. The Ag growth induced by PMCRs is also observed near the synaptic nanostructures, growing into a honeycomb-like pattern due to the limited material transfer between the neighboring PS beads. The nanohoneycomb array shows significant coupling between the side wall and the Ag nanocap at the center, which is suitable for a new SERS-active substrate for the detection of thiram pesticides down to 10^−9^ M. This study advocates for quick and effective pesticide residue detection.

## Data Availability

No new data.
